# Patient-reported symptom burden in patients with rare cancers receiving pembrolizumab in a phase II Clinical Trial

**DOI:** 10.1038/s41598-022-16588-3

**Published:** 2022-08-23

**Authors:** Tito R. Mendoza, David S. Hong, Christine B. Peterson, Bettzy Stephen, Ecaterina Dumbrava, Shubbam Pant, Apostolia Maria Tsimberidou, Timothy Anthony Yap, Ajay Sheshadri, Mehmet Altan, Goldy George, Lilibeth Castillo, Enedelia Rodriguez, Jing Gong, Vivek Subbiah, Filip Janku, Siqing Fu, Sarina A. Piha-Paul, Jordi Rodon Ahnert, Daniel D. Karp, Charles Cleeland, Funda Meric-Bernstam, Aung Naing

**Affiliations:** 1 Department of Symptom Research, The University of Texas MD Anderson Cancer Center, Houston, TX USA; 2 Department of Investigational Cancer Therapeutics, The University of Texas MD Anderson Cancer Center, Houston, TX USA; 3 Department of Biostatistics, The University of Texas MD Anderson Cancer Center, Houston, TX USA; 4 Department of Pulmonary Medicine, The University of Texas MD Anderson Cancer Center, Houston, TX USA; 5 Department of Thoracic/Head and Neck Medical Oncology, The University of Texas MD Anderson Cancer Center, Houston, TX USA

**Keywords:** Cancer immunotherapy, Signs and symptoms

## Abstract

Patients with rare solid tumors treated on early phase trials experience toxicities from their tumors and treatments. However, limited data exist to describe the detailed symptom burden suffered by these patients, particularly those with rare solid tumors treated with immunotherapy. We performed a prospective longitudinal study to capture patient-reported symptom burden. Patients completed the validated MD Anderson Symptom Inventory (MDASI)—Immunotherapy with 20 symptoms including 7 immunotherapy-specific items and 6 interference items at baseline and weekly thereafter for up to 9 weeks. Symptoms and interference were rated on 0–10 scales (0 = none or no interference, 10 = worst imaginable or complete interference). Group-based trajectory modelling determined higher and lower symptom groups. A total of 336 MDASI questionnaires were completed by 53 patients (mean age 55.4y, 53% male) with advanced rare cancers receiving pembrolizumab in a Phase II clinical trial. Symptoms reported as most severe over the course of the treatment over 9 weeks were fatigue [mean (M) = 3.8, SD = 2.3], pain (M = 3.7, SD = 2.9), disturbed sleep (M = 2.7, SD = 2.3), drowsiness (M = 2.6, SD = 2.0) and lack of appetite (M = 2.5, SD = 2.1). Pain in the abdomen (M = 2.2, SD = 2.4), rash (M = 1.1, SD = 1.8) and diarrhea (M = 0.9, SD = 1.5) were less severe. Interference with walking was rated the highest (M = 3.4, SD = 2.8) and relations with others was rated the lowest (M = 2.1, SD = 2.6). Using a composite score based on the five most severe symptoms (fatigue, pain, lack of appetite, feeling drowsy and sleep disturbance), 43% were classified into the high symptom burden group. Using a score based on immunotherapy-specific symptoms (e.g., rash, diarrhea) 33% of patients were included in the high symptom group. Symptom burden stayed relatively stable in the high- and low-symptom burden patient groups from baseline through 9 weeks. Some patients with rare malignancies experienced high symptom burden even at baseline. In patients with rare cancers, symptom trajectories stayed relatively stable over nine weeks of treatment with pembrolizumab.

Trial registration: ClinicalTrials.gov identifier: NCT02721732.

## Introduction

Patient reported outcomes (PRO) provide a patient the opportunity to describe what a patient is experiencing during and after treatment, the degree to which treatment produces such symptoms, and impact on functioning. The use of validated PRO measures in clinical trials can provide the patient’s assessment of the severity and impact of treatment-related symptoms of new therapies. This assessment provides critical information to patients, providers, regulators, and third-party payers for evaluating the tolerability of these therapies^1^ and for judging the safety and value (ratio of treatment benefit relative to cost)^2^ of one therapy over another. This is especially true when new therapies provide only small increases in overall survival or time to progression, or are effective for only a modest percentage of the patients who receive them. Thus, PROs may be considered an essential component of oncology drug development, without which clinicians and regulators have an incomplete picture of how patients are affected by a new agent^3^. Indeed, in a policy review, international regulatory agencies from the USA, Europe, and Canada have acknowledged interest in the inclusion of PROs throughout the drug development process^4^. Most symptomatic toxicities data currently are from clinician-registered adverse event (AE) reports, and prior research by others suggest that symptomatic AEs are likely to be underreported by clinicians^5^.

Immune checkpoint inhibitors are a new class of immunotherapeutic agents that remove the inhibitory signal provided to immune T cells so they can launch a cytotoxic attack on tumor cells. It is increasingly clear that immune checkpoint inhibitors produce a unique and emerging set of irAEs^6,7^, many of which present as symptoms and can thus be well captured by patient report. Because irAEs are classically autoimmune in nature^8^ and are often T-cell-mediated^9^, the toxicities of checkpoint inhibitors may be caused by T cells indiscriminately attacking both tumor cells and normal cells. Given the effects of checkpoint inhibitors on the immune system, their side-effect profiles may differ somewhat from those of other cancer therapies, including conventional cytotoxic chemotherapy. Yet, although immune checkpoint inhibitors seem promising in prolonging survival, little is known about their symptom related benefit or burdens from the patient’s perspective. As immunotherapy become generally used, it is important to be able to describe to patients what types of side effects they could expect from the treatment^10^. While there are trials that have used PRO, few have used frequent assessments to describe the trajectory of symptoms longitudinally across treatment in patients with rare cancers.

Pembrolizumab is an immune checkpoint inhibitor that blocks the programmed cell death protein 1 (PD1), an inhibitory receptor expressed by immune T cells. PD1 blockade enables T cells to destroy cancer cells. Pembrolizumab was granted accelerated approval by the FDA not only for treatment of multiple tumor types, but also for tumor-agnostic conditions such as high microsatellite instability (MSI-H) or mismatch repair deficient (dMMR) solid tumors and those with high tumor mutational burden^11^. We have previously reported clinical activities of Pembrolizumab in patients with advanced rare tumors^12^.

We report here frequent assessment of symptomatic status of patients in a Phase II trial of patients with rare cancer receiving pembrolizumab. We describe the symptom patterns of these patients and classified them into two groups based on the severity of their longitudinal symptom severity. We elucidate how these patients feel and function with regards to their immune checkpoint inhibitor treatment.

## Methods

### Study participants

Ten patient cohorts with advanced rare cancers were used to describe the symptom burden associated with pembrolizumab. Patients completed the validated MD Anderson Symptom Inventory—Immunotherapy (MDASI-Immunotherapy EPT) module for early phase trials (13) at baseline and weekly thereafter for up to 9 weeks. Symptoms and interference were rated on 0–10 scales (0 = none or no interference, 10 = worst imaginable severity or complete interference).

Patients were in a Phase II basket trial in the Clinical Center for Targeted Therapy clinic at MD Anderson and were receiving 200 mg of pembrolizumab intravenously every three weeks. (12) Sample size for this descriptive longitudinal study was based on reporting the symptom results with reasonable confidence limits using eligible patients. To be eligible for this PRO-based study, patients were required to be at least 18 years old, speak English, and have a pathological diagnosis of a rare cancer, whose disease had progressed while on standard therapies (if available) within the previous 6 months. Patients were excluded if clinical research staff felt that they did not understand the intent of the study or could not complete the assessment measures. This study was approved by the Institutional Review Board of The University of Texas MD Anderson Cancer Center in Houston, Texas (MDACC protocols 2015–0948 and PA15-0315). The study was conducted in accordance with the Declaration of Helsinki and the International Conference on Harmonization Good Clinical Practice guidelines. All the study participants provided written informed consent before enrollment.

### Demographic and clinical data

At the time of patient enrollment, research staff asked study participants to complete self-administered questionnaires, answered questions, and assisted with completion of survey forms as needed. Patient demographic information (e.g., sex, age, marital status, education level, and employment status) were collected during the initial clinic visit.

Medical background information was extracted from electronic medical records, including cancer diagnosis, and Eastern Cooperative Oncology Group performance status (ECOG PS). ECOG PS^14^ was used to estimate disease severity and is a physician-rated measure of functional ability, ranging from 0 (fully active; able to carry on all pre-disease performance without restriction) to 4 (completely disabled; cannot perform self-care; totally confined to bed or chair).

### Study instrument

#### The M. D. Anderson Symptom Inventory – Immunotherapy for early phase trials (EPT)

The MDASI-Immunotherapy EPT has 20 symptoms including 7 immunotherapy-specific items in addition to the 13 MDASI core symptoms^15^. The MDASI asks patients to rate the severity of disease-related and treatment-related symptoms during the past 24 h. Each symptom (pain, fatigue, nausea, disturbed sleep, emotional distress, shortness of breath, difficulty remembering, lack of appetite, drowsiness, dry mouth, sadness, vomiting, numbness or tingling, rash, diarrhea, pain the abdomen, swelling in the hands and legs, headache, night sweats and fever and/or chills) is rated on an 11-point scale ranging from 0 (not present) to 10 (as bad as you can imagine). The MDASI-Immunotherapy EPT has been shown to be valid and reliable^13^.

Patients also rate the degree to which symptoms interfered with various aspects of life during the past 24 h. Each interference item (general activity, mood, normal work [including both work outside the home and housework], relations with other people, walking ability, and enjoyment of life) is rated on an 11-point scale ranging from 0 (did not interfere) to 10 (interfered completely). The interference factor can be decomposed into (1) an activity-related interference dimension consisting of the items normal work, general activity, and walking ability, and (2) a mood-related interference dimension composed of the items relations with people, enjoyment of life, and mood^16^.

### Statistical analysis

Most statistical analyses were conducted using Statistical Package of the Social Sciences (SPSS) software version 26. Correlations, means, standard deviations (SDs), ranges, and 95% confidence limits (CL) were computed for all symptoms and subscales. Statistical significance was set using a two-tailed alpha level of 0.05.

#### Group-based trajectory modeling (GBTM)

Cluster analysis is a commonly used method to group patients based on how similarly they report the severity of their symptoms. But because symptoms change over the course of cancer treatment, patient groupings may vary across time points. To account for this, we used group-based trajectory model (GBTM) under PROC TRAJ in SAS. This method is a statistical approach designed to group longitudinal observations into interrelated subgroups. Like cross-sectional methods, GBTM takes into consideration measures at a given time point, but unlike cross sectional methods, GBTM considers the change patterns of those measures across multiple time points. GBTM may identify two or more groups with distinct trajectories. For simplicity and because of our limited sample size, we sought to determine group membership in either high or low symptom burden over 9 weeks depending on the outcome variable used. In our case, we performed GBTM using two outcome variables. First, we created high and low symptom burden groups based on the average of the top 5 symptoms namely, fatigue, pain, lack of appetite, feeling drowsy and disturbed sleep. Second, we used the average of the 7 immunotherapy-specific symptoms to form groups. The group trajectories were described as linear regression lines with intercept and slope. A non-significant slope estimate suggests a stable pattern.

## Results

### Demographic and clinical characteristics of study cohorts

If all 53 patients completed every MDASI assessments from baseline to the end of 9 weeks, then we will have 530 MDASIs. Because some patients dropped out of the study, no further MDASI assessments were expected for those patients. Out of the 502 expected MDASI questionnaires, 336 were completed by 53 patients for a 67% completion rate. Reasons for dropout/termination included non-compliance (n = 7), progression (n = 20), withdrawal of consent (n = 2), and toxicity (n = 2). Demographic and clinical characteristics of the patient cohort of 53 patients are summarized in Table 1. Overall, the average and median age were 55.4 and 61 years respectively, and the mean education level was 14 years. Male represented 53% (n = 28), and the sample was predominantly non-Hispanic white. Most patients had an ECOG performance status of 1.

**Table 1:** Demographic and clinical characteristics of study cohorts (*n* = 53).

Patient characteristics	Mean (SD)
Age, years	55.4 (17.9)
Education level, years	14 (2.6)

	N (%)
**Sex**	
Men	28 (53)
Women	25 (47)
**Race**	
Black or African-American	4 (7)
White	39 (74)
Asian	3 (6)
Other	6 (11)
Unknown	1 (2)
**Hispanic or Latino ethnicity**	
Hispanic or Latino	8 (15)
Non-Hispanic	44 (83)
Unknown	1 (2)
**ECOG PS score at baseline**	
0	6 (11)
1	47 (89)
**Tumor Types**	
Squamous cell carcinoma of the skin	8 (15)
Small cell malignancies of non-pulmonary origin—cervical, prostate, vulva	6 (11)
Carcinoma of unknown primary	7 (13)
Vascular sarcoma	4 (8)
Paraganglioma pheochromocytoma	4 (8)
Adrenocortical carcinoma	2 (4)
Medullary renal cell carcinoma	2 (4)
Penile carcinoma	2 (4)
Testicular carcinoma/ germ cell tumor	3 (5)
Other rare histologies	15 (28)

Abbreviations: MDASI-Immunotherapy, immunotherapy module of the M. D. Anderson Symptom Inventory; SD, standard deviation; ECOG PS, Eastern Cooperative Oncology Group performance status.

### Pattern of symptom burden during the first 9 weeks of pembrolizumab treatment

Table 2 shows symptom severity in patients with rare solid tumors at baseline, at weeks 1–3, at weeks 4–6, weeks 7–9, and the average of values for 9 weeks of treatment with pembrolizumab. Symptoms with the worst severity at baseline were fatigue, pain, disturbed sleep, lack of appetite and drowsiness. The most severe symptoms at 7–9 weeks of treatment with pembrolizumab were pain, fatigue, lack of appetite, disturbed sleep and drowsiness. Symptoms reported as most severe over the course of the treatment over 9 weeks were fatigue [mean (M) = 3.8, SD = 2.3), pain (M = 3.7, SD = 2.9), disturbed sleep (M = 2.7, SD = 2.3), feeling drowsy (M = 2.6, SD = 2.0) and lack of appetite (M = 2.5, SD = 2.1). Pain in the abdomen (M = 2.2, SD = 2.4), rash (M = 1.1, SD = 1.8) and diarrhea (M = 0.9, SD = 1.5) were rated lower and ranked as 6^th^, 16.5^th^ and 18^th^ worst of the 20 symptoms. Interference with walking was rated the highest (M = 3.4, SD = 2.8) with relations rated as the lowest (M = 2.1, SD = 2.6).

**Table 2:** Mean (SD) symptom severity^*^ at baseline, at weeks 1–3, at weeks 4–6, weeks 7–9, and overall from baseline to week 9 of treatment with pembrolizumab in patients with rare solid tumors.

Symptom at baseline	Baseline (week 0) Mean (SD) N = 53	Baseline rank*	Weeks 1–3 mean (SD) N = 46	Weeks 1–3 rank*	Weeks 4–6 mean (SD) N = 39	Weeks 4–6 rank*	Weeks 7–9 mean (SD) N = 34	Weeks 7–9 rank*	Overall up to 9 weeks mean** (SD)	Overall up to 9 weeks rank**
Pain	3.7 (3.3)	1.5	3.6 (3.2)	2	3.9 (3.0)	2	3.7 (3.3)	1.5	3.7 (2.9)	2
Fatigue	3.7 (3.1)	1.5	4.0 (2.6)	1	4.1 (2.6)	1	3.7 (2.4)	1.5	3.8 (2.3)	1
Disturbed sleep	2.7 (2.6)	3	2.6 (2.6)	3.5	2.8 (2.9)	4	2.7 (2.3)	4	2.7 (2.3)	3
Lack of appetite	2.6 (3.1)	4	2.2 (2.3)	5.5	2.6 (2.4)	5.5	2.9 (2.7)	3	2.5 (2.1)	5
Drowsy	2.5 (2.3)	5	2.6 (2.5)	3.5	3.0 (2.6)	3	2.6 (2.3)	5	2.6 (2.0)	4
Distress	2.3 (2.6)	6	1.8 (2.3)	8.3	1.6 (2.1)	10	1.7 (2.2)	9	2.0 (2.2)	8.3
Numbness	2.1 (2.5)	7.5	1.8 (2.4)	8.3	2.1 (2.7)	8	2.2 (2.8)	6.5	2.0 (2.3)	8.3
Shortness of breath	2.1 (2.6)	7.5	1.8 (2.3)	8.3	1.9 (2.0)	9	1.5 (1.6)	12.5	2.0 (2.1)	8.3
Pain in the abdomen	1.9 (2.6)	9	2.2 (2.6)	5.5	2.3 (2.6)	7	2.1 (2.5)	8	2.2 (2.4)	6
Sadness	1.7 (2.3)	10.5	1.5 (2.0)	12	1.4 (1.9)	12.3	1.6 (2.2)	10.5	1.7 (2.0)	11
Nausea	1.7 (3.1)	10.5	0.9 (1.4)	17	1.1 (2.0)	16.5	1.2 (1.8)	16	1.3 (1.8)	14
Dry mouth	1.6 (2.1)	12.3	2.1 (2.7)	7	2.6 (2.7)	5.5	2.2 (2.4)	6.5	2.1 (2.2)	7
Difficulty remembering	1.6 (2.0)	12.3	1.6 (2.0)	11	1.5 (1.9)	11	1.6 (1.6)	10.5	1.6 (1.8)	12
Swelling	1.6 (2.7)	12.3	1.2 (2.2)	14.5	1.4 (2.3)	12.3	1.4 (2.3)	14.5	1.5 (2.5)	13
Diarrhea	1.1 (2.4)	15.5	0.8 (1.2)	18	0.5 (1.0)	19	0.5 (1.1)	19.5	0.9 (1.5)	18
Headache	1.1 (2.2)	15.5	1.2 (2.5)	14.5	1.1 (2.3)	16.5	1.0 (2.1)	17	1.1 (2.0)	16.5
Night sweat	0.9 (1.3)	17.5	1.1 (1.7)	16	1.4 (1.4)	12.3	1.4 (1.8)	14.5	1.2 (1.4)	15
Rash	0.9 (1.8)	17.5	1.3 (2.1)	13	1.3 (2.3)	15	1.5 (2.4)	12.5	1.1 (1.8)	16.5
Vomiting	0.7 (2.1)	19	0.4 (1.0)	20	0.3 (0.9)	20	0.5 (1.2)	19.5	0.5 (0.9)	20
Fever or chills	0.6 (1.4)	20	0.6 (1.2)	19	0.6 (1.3)	18	0.7 (1.3)	18	0.6 (1.2)	19

*Symptoms at each time (at baseline, at weeks 1–3, at weeks 4–6, weeks 7–9, and overall from baseline to 9 weeks of treatment) are ranked at that respective time point.

**For the calculation of the overall mean of symptom severity for each symptom over the entire treatment course from baseline to 9 weeks, the aggregated mean for each patient was first calculated prior to computing the means for the overall sample.

Aggregated means were obtained for each patient prior to calculating the symptom severity at each specific time point for all patients for whom data were available at that time point.

**Figure 1:**
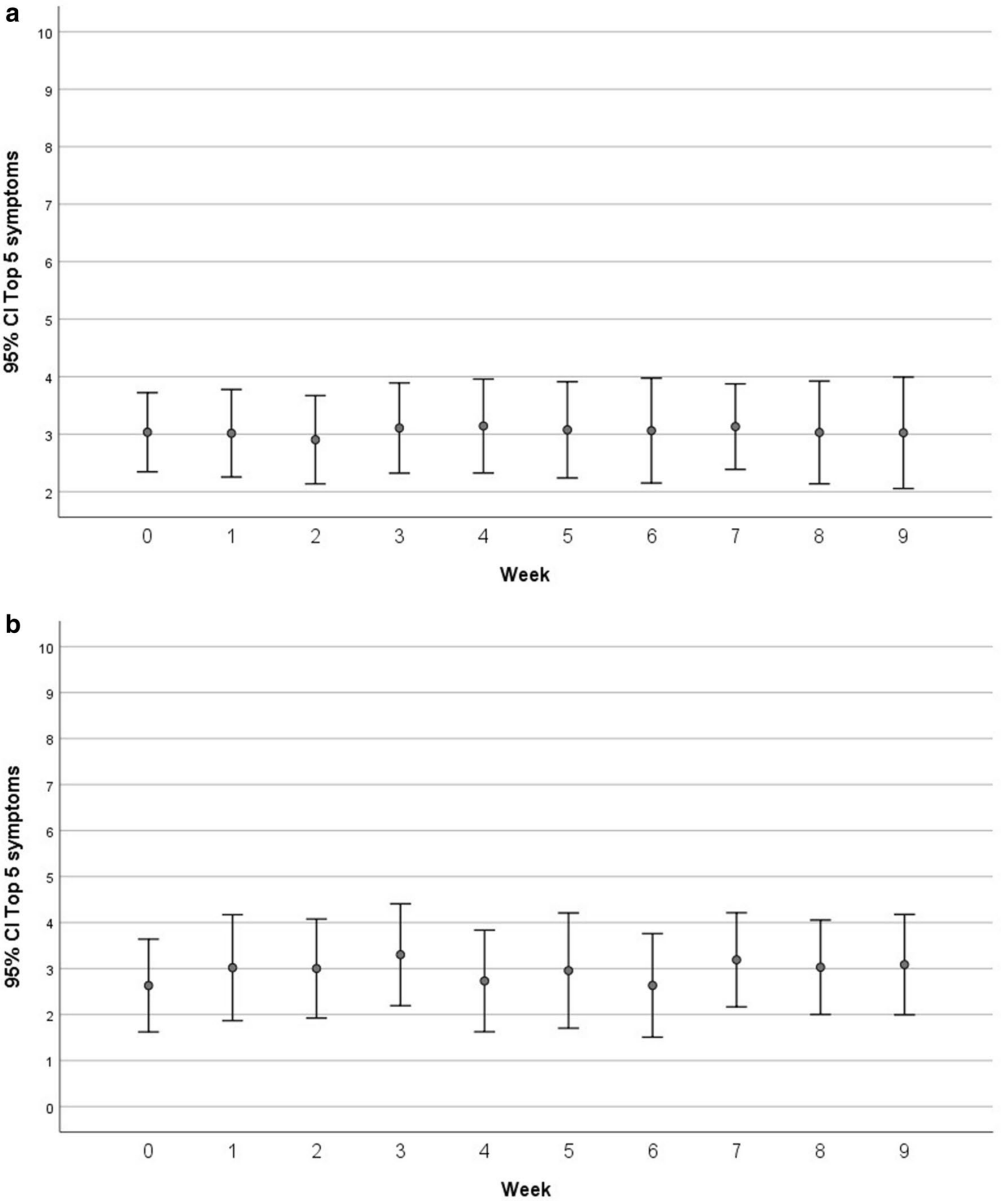
(**a**) Mean values for the composite score of the top five symptoms (pain, fatigue, disturbed sleep, lack of appetite, and drowsiness) over 9 weeks of treatment with pembrolizumab in all patients with rare tumors who started the trial (N = 53). (**b**) Mean values for the composite score of the top five symptoms over 9 weeks of treatment with pembrolizumab in patients with rare tumors who completed 9 weeks of treatment with pembrolizumab (N = 22).

Figure 1a shows relative stability of a composite score of the top five symptoms (albeit with very minor variations) over 9 weeks of treatment with pembrolizumab using all available data at each time points. In order to determine whether dropouts affected the severity of symptom patterns, we reproduced the plot in Fig. 1a but using data only from patients with complete data for all 9 weeks. Figure 1b is very similar to Fig. 1a in that the mean severity is around 3 on a 0 to 10 scale.

**Figure 2:**
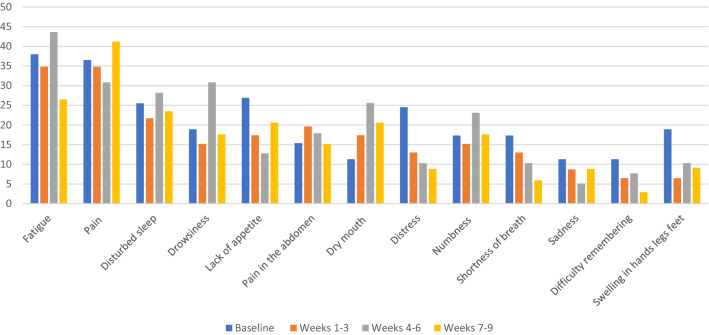
Percentage of patients with moderate-to-severe severity (MDASI scores greater than or equal to 5 on a 0–10 scale) of top 13 symptoms at baseline, weeks 1–3, weeks 4–6, and weeks 7–9 of treatment with pembrolizumab.

Figure 2 shows the percentages of patients experiencing moderate to severe levels of the 13 most prominent symptoms over the 9 weeks grouped into 4 time periods of pembrolizumab treatment. More than a third of patients experience fatigue at the beginning of their treatment with a quarter of the patients still reporting fatigue to be moderate to severe. At least 10% of patients reported moderate to severe levels at baseline for any of the top 13 symptoms. With the exception of pain and dry mouth, patients reported that many symptoms either stayed the same or showed slight improvement from baseline to Weeks 7–9.

### Group-based trajectory pattern of symptom burden during the first 9 weeks of pembrolizumab treatment

**Figure 3:**
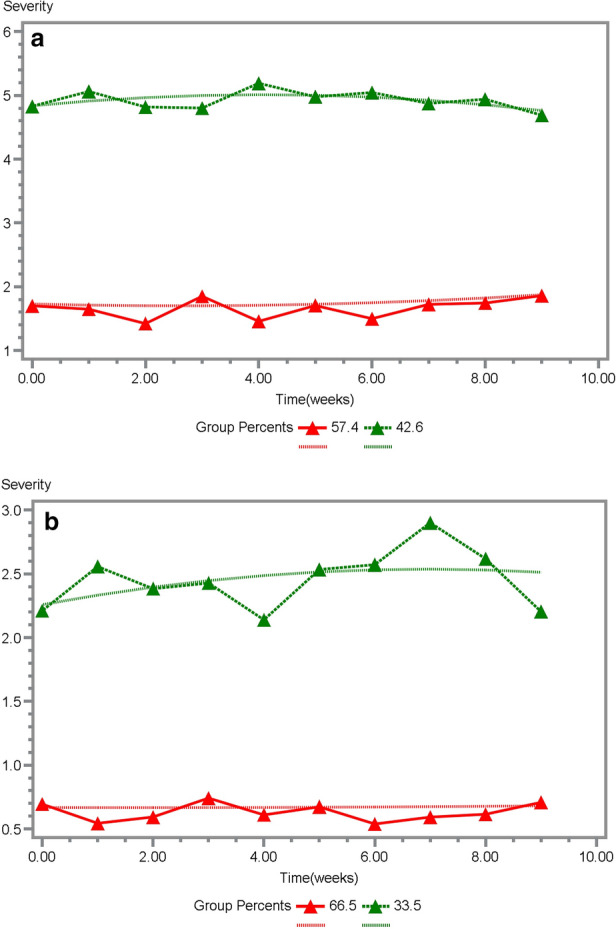
(**a**) Group-based trajectory modeling of the top 5 symptoms. (**b**) Group-based trajectory modeling of the immunotherapy specific symptoms.

Figure 3a shows the results of group- based trajectory modeling of the top 5 symptoms. Using a composite score based on the five most severe symptoms (pain, fatigue, lack of appetite, sleep disturbance and feeling drowsy), 43% were classified into the high symptom burden group. There were no significant increase in symptom during this period for either the high symptom burden group (est = − 0.02, *p* < 0.87) or the low burden symptom group (est = 0.08, *p* < 0.65). Figure 3b presents the group-based trajectory modeling using a score based on immunotherapy-specific symptoms (e.g., rash, diarrhea). It resulted in 33% of patients belonging to the high symptom group. Similarly, there were no significant increase in symptom during this period for either the high symptom burden group (est = − 0.001, *p* < 0.98) or the low burden symptom group (est = 0.08, *p* < 0.43). Symptom trajectories are relatively stable over 9 weeks of treatment with pembrolizumab. Patients with high symptom burden at baseline continued to have high symptom burden during treatment with pembrolizumab, and patients with low symptom burden at baseline entry into the clinical trial continue to have low symptom burden over the course of 9 weeks of treatment with pembrolizumab.

## Discussion

To our knowledge, the present study is the first to examine longitudinally patient-reported symptoms in patients with rare cancers in a clinical trial testing an immune checkpoint inhibitor. Although patient-reported outcomes have been included in immunotherapy trials, this study provides a very frequent (every week) examination of symptom burden associated with immunotherapy compared to assessments done mostly at imaging visits (every 8–9 weeks). Knowing the symptom trajectories associated with the use of immunotherapy contributes to our understanding of symptom benefit and whether patients can continue with their cancer treatment. Tolerability of treatment using patient-reported outcomes is an important component of drug evaluation^1^.

The finding in the present study that symptom trajectories stayed relatively stable over 9 weeks of treatment with pembrolizumab is parallel to observations by Lacey et al.^17^ that treatment with pembrolizumab in patients with metastatic melanoma was accompanied by low symptom burden and that changes in symptoms longitudinally were not seen regardless of whether or not patients’ usual care was supplemented with a supportive care intervention. However, the KEYNOTE-087 study in patients with relapsed/refractory classical Hodgkin lymphoma who received pembrolizumab as monotherapy reported improvements in overall quality of life from baseline to weeks 12–24 of treatment with pembrolizumab^18^. The KEYNOTE-087 study also showed improvements during treatment with pembrolizumab in symptoms assessed (fatigue, nausea and vomiting, pain, dyspnea, insomnia, constipation and diarrhea). Also, a study by Barlesi et al.^19^ in patients with advanced non-small cell lung cancer (NSCLC) receiving pembrolizumab or docetaxel in a randomized trial found that pembrolizumab was linked to better quality of life scores than docetaxel. Barlesi et al.^19^ reported nominally significant improvement in many EORTC-QLQC30 symptom domains with pembrolizumab, and nominally significant worsening with docetaxel.

The strengths of the present study include the unique study population of patients with rare cancers participating in a Phase II clinical trial, and the systematic and longitudinal collection of patient-reported outcomes data focused on symptoms in a clinical trial testing an immune checkpoint inhibitor, and that all patients in this Phase II trial received the same dose of pembrolizumab. Weaknesses include a relatively small sample size of 53 patients, and a relatively brief follow-up period of 9 weeks. Yet, this study offers valuable insights into weekly patient-reported symptoms during the first nine weeks of treatment with pembrolizumab in patients with rare tumors. Although symptoms are stable over the first nine weeks, our group-based trajectory analyses showed that there are subgroups of patients who have consistently high symptom burden. The proportion of patients experiencing high symptom burden changes depending on the type of symptoms. For this study, it was 43% based on pain, fatigue, lack of appetite, sleep disturbance and feeling drowsy but was only 33% based on immunotherapy specific symptoms such as rash and diarrhea. Our examination of associations of baseline demographic and clinical characteristics with symptom burden grouping yielded no significant results. Similarly, our examination of differences in symptom ratings by treatment response (objective response: yes/no) resulted in no significant result. Because of the limited sample size, these analyses were inconclusive. Future studies with larger sample sizes can identify demographic and clinical characteristics of those patients with high symptom burden that may allow personalized symptom management.

In summary, our results suggest it is feasible to collect PRO data in patients with rare cancers enrolled in a Phase II trial of an immune checkpoint inhibitor. Our findings indicate that symptom trajectories in patients with rare cancers receiving pembrolizumab remain relatively stable over the first 9 weeks of treatment and that baseline symptom groupings are associated with the trajectory of symptom burden over 9 weeks of treatment with pembrolizumab as monotherapy. Our results may inform clinicians about symptom intervention needs, and provide a benchmark in designing future symptom intervention clinical trial. These findings need to be investigated further in larger samples with longer duration of follow-up.

## Data Availability

The datasets used and/or analyzed during the current study are available from the corresponding author on reasonable request according to available guidelines at the time of request.

## References

[1] Kluetz PG, Kanapuru B, Lemery S, Johnson LL, Fiero MH, Arscott K (2018). Informing the tolerability of cancer treatments using patient-reported outcome measures: Summary of an FDA and critical path institute workshop. Value Health..

[2] Schnipper LE, Davidson NE, Wollins DS, Tyne C, Blayney DW, Blum D (2015). American society of clinical oncology statement: A conceptual framework to assess the value of cancer treatment options. J. Clin. Oncol..

[3] Basch E (2018). Patient-reported outcomes: an essential component of oncology drug development and regulatory review. Lancet Oncol..

[4] Kluetz PG, O'Connor DJ, Soltys K (2018). Incorporating the patient experience into regulatory decision making in the USA, Europe, and Canada. Lancet Oncol..

[5] Di Maio M, Basch E, Bryce J, Perrone F (2016). Patient-reported outcomes in the evaluation of toxicity of anticancer treatments. Nat. Rev. Clin. Oncol..

[6] Mendoza TR (2018). Symptoms as patient-reported outcomes in cancer patients undergoing immunotherapies. Immunotherapy.

[7] Naing A (2018). Being realistic and optimistic in curing cancer. J. Immunother. Precis. Oncol..

[8] Socinski MA (2015). Incorporating immunotherapy into the treatment of non-small cell lung cancer: Practical guidance for the clinic. Semin Oncol..

[9] Weber JS, Yang JC, Atkins MB, Disis ML (2015). Toxicities of immunotherapy for the practitioner. J. Clin. Oncol..

[10] Naing A, Hajjar J, Gulley JL, Atkins MB, Ciliberto G, Meric-Bernstam F (2020). Strategies for improving the management of immune-related adverse events. J. Immunother. Cancer..

[11] Marabelle A, Le DT, Ascierto PA, Di Giacomo AM, De Jesus-Acosta A, Delord JP (2020). Efficacy of pembrolizumab in patients with noncolorectal high microsatellite instability/mismatch repair-deficient cancer: Results from the phase II KEYNOTE-158 study. J. Clin. Oncol..

[12] Naing, A., Meric-Bernstam, F., Stephen, B., Karp, D. D., Hajjar, J., Ahnert, J. R., & Habra, M. A. Phase 2 study of pembrolizumab in patients with advanced rare cancers. *J. Immunother. Cancer* **8**(1) (2020). 10.1136/jitc-2019-000347 PMC7078933 32188704

[13] Mendoza T, Sheshadri A, Altan M, Hess K, George G, Stephen B (2020). Evaluating the psychometric properties of the immunotherapy module of the MD anderson symptom inventory. J. Immunother. Cancer..

[14] Oken MM, Creech RH, Tormey DC, Horton J, Davis TE, McFadden ET (1982). Toxicity and response criteria of the eastern cooperative oncology group. Am. J. Clin. Oncol..

[15] Cleeland CS, Mendoza TR, Wang XS, Chou C, Harle MT, Morrissey M (2000). Assessing symptom distress in cancer patients: The M.D. anderson symptom inventory. Cancer.

[16] Cleeland CS, Nakamura Y, Mendoza TR, Edwards KR, Douglas J, Serlin RC (1996). Dimensions of the impact of cancer pain in a four country sample: New information from multidimensional scaling. Pain.

[17] Lacey J, Lomax AJ, McNeil C, Marthick M, Levy D, Kao S (2019). A supportive care intervention for people with metastatic melanoma being treated with immunotherapy: A pilot study assessing feasibility, perceived benefit, and acceptability. Support. Care Cancer.

[18] von Tresckow B, Fanale M, Ardeshna KM, Chen R, Meissner J, Morschhauser F (2019). Patient-reported outcomes in KEYNOTE-087, a phase 2 study of pembrolizumab in patients with classical Hodgkin lymphoma. Leuk. Lymphoma.

[19] Barlesi F, Garon EB, Kim DW, Felip E, Han JY, Kim JH (2019). Health-Related quality of life in KEYNOTE-010: A phase II/III study of pembrolizumab versus docetaxel in patients with previously treated advanced, programmed death ligand 1-expressing NSCLC. J. Thorac. Oncol..

